# Fault Diagnosis for Abnormal Wear of Rolling Element Bearing Fusing Oil Debris Monitoring

**DOI:** 10.3390/s23073402

**Published:** 2023-03-23

**Authors:** Yulai Zhao, Xiaowei Wang, Shuo Han, Junzhe Lin, Qingkai Han

**Affiliations:** 1 School of Mechanical Engineering and Automation, Northeastern University, Shenyang 110819, China; 2 Key Laboratory of Vibration and Control of Aero-Propulsion System Ministry of Education, Northeastern University, Shenyang 110819, China

**Keywords:** rolling element bearing, abrasive wear, neighborhood component analysis, oil debris, fault diagnosis

## Abstract

The abnormal wear of a rolling element bearing caused by early failures, such as pitting and spalling, will deteriorate the running state and reduce the life. This paper demonstrates the importance of oil debris monitoring and its effective feature extraction for bearing health assessment. In this paper, a rolling bearing-rotor test rig with forced lubrication is set up and the nonferrous contaminants with higher hardness were introduced artificially to accelerate the occurrence of pitting and spalling. The early failure and abnormal wear of rolling bearings cannot be effectively detected only through the vibration signal; the temperature and oil debris monitoring data are also collected synchronously. Two features regarding the ferrous particle size distribution are extracted and fused with vibration based-features to form a feature set. The sensitive features are extracted from the features set using the Neighborhood Component Analysis method to avoid feature redundancy. Finally, the importance of the oil debris based-features for the diagnosis of abnormal bearing wear is analyzed with different machine learning algorithms. Taking SVM classifier as an example, the experiment results show that the introduction of oil debris based-features increases the diagnostic accuracy by 15.7%.

## 1. Introduction

Rolling element bearings are important parts of rotating machinery, such as aero-engines and compressors. The service status of bearings significantly affects the performance of the machines. Affected by rotor unbalance, poor lubrication, contaminant, manufacturing defects, etc., the bearing is prone to abnormal wear caused by contact surfaces pitting or even spalling. The spalled ferrous particles will continue to aggravate bearing wear, and eventually deteriorate the bearing service environment or scrap the bearing. Therefore, using various measurements, signal processes, and fault diagnosis methods to explore the abnormal wear mechanism and evaluate the health status of the bearing is essential for the long-term stable operation of rotating machinery [1].

At present, the monitoring methods for rolling element bearings mainly include temperature, vibration acceleration, rotating shaft whirl, oil/grease, thermal imaging, acoustic emission, etc. The measurement of bearing temperature with a thermocouple and temperature distribution with thermal imaging are two commonly used methods [2,3]. Fiber Bragg Grating (FBG) sensors and fiber optic rotary joints are used to test the bearing rings multi-point temperature in Ref. [2], and the influence of load and speed on the ring temperature is analyzed. The relationship between bearing temperature and bearing dynamic stiffness is studied in Ref. [3]. The results show that during the warm-up, the bearing stiffness shows strong nonlinearity and time-varying characteristics. Mohanty and Fatima [4] used thermal imaging technology to detect misalignment faults in a bearing-rotor system and analyzed the effects of load, speed, and coupling type on the detection results. Temperature monitoring can effectively avoid the occurrence of serious bearing failures. However, in the early stage of bearing service, the temperature change of the bearing is not obvious. Therefore, temperature monitoring can only be used as an assistant.

Abnormal wear will accelerate the bearing to produce pitting or even spalling, and then generate ferrous particles in lubricating oil/grease. By monitoring the size and material of the particles, the wear mechanism can be revealed, and the severity and location of failure can also be effectively judged. The monitoring of rolling element bearing based on oil debris monitoring (ODM) is more direct and can predict wear in advance, especially for bearing early wear and abnormal wear [5]. In this paper, nonferrous contaminants with higher hardness are introduced into the bearing to accelerate the occurrence of pitting or spalling. The influence of the size and hardness of the contaminants on bearing wear is explored in Ref. [6]. The extraction of features from the online oil debris monitoring data that can effectively differentiate the bearing wear state is the focus of the current research [7]. Loutas [8] proposed to monitor gear fatigue wear failure using ferrous particle mass and mass rate combined with vibration based-features. Kumar [9] proposed to use the size distribution to monitor the fatigue failure of bearings, but the analysis is qualitative, not quantitative. In addition, there are few studies focused on size distribution relative features to difference bearing abnormal wear status.

Ranjan [10] found an abnormal increase in the aspect ratio of the wear particles in the later stage of journal bearing service by monitoring the oil debris. Akagaki [11] analyzed the relationship between oil debris contaminants and the friction coefficient, wear rate, and vibration characteristics of rolling bearings; the oil debris detection method is offline. Craig [12] improved the accuracy of bearing damage diagnosis by combining the online oil debris monitoring method with the offline detection method. The development of high-precision oil debris sensors is also an important prerequisite for improving the accuracy of bearing diagnosis [13,14]. Liu [15] proposed an online optical oil debris sensor. The experimental results show that the measurement error of debris density and oil viscosity is only 6.07% and 7.97%, respectively.

The direct manifestation of bearing wear is mass loss, which can be directly obtained by continuous oil debris monitoring. Although the importance of oil debris monitoring for bearing wear fault diagnosis is well known, the specific contribution of oil debris monitoring compared with only relying on vibration sensors is unknown. In order to solve this problem, this paper quantitatively analyzes the influence of fusing oil debris and vibration signal and only relies on the vibration signal on bearing diagnosis through bearing wear experiments.

Due to simple and convenient data collection and mature signal analysis methods, bearing fault diagnosis with vibration signal-based techniques is widely used [16]. When the rolling element passes through the damaged area, the bearing will produce an obvious impact signal [17]. The commonly used signal analysis methods include envelope spectrum, cepstrum, spectrum kurtosis, EMD, wavelet transform, and statistics-based methods [18,19,20,21,22]. Based on resonance theory, SKF proposed the shock pulse method (SPM) for the fault diagnosis of bearings [23,24]. However, for early bearing wear, the vibration signal of the bearing lacks obvious fault characteristics [25,26]. The bearing wear cannot be detected effectively only by the vibration signal [27]. Aiming at the insufficiency of a single type of sensor, the fault diagnosis method based on multi-sensors has been gradually developed [28]. Hiremath and Reddy [29] judge the wear of the outer race of the roller bearing through the degradation of grease and analysis of vibration signals and temperature. Safizadeh and Golmohammadi [30] use multi-sensor data to detect the pressure distribution change of journal bearing caused by oil whirl.

Although the above research adopts multi-physical quantity monitoring, it does not analyze the relationship between various physical quantities and key sensitive features reflecting the system state [31,32]. Based on this, this paper adopts multi-sensor monitoring methods, including vibration, temperature, and oil debris. Meanwhile, the Neighborhood Component Analysis (NCA) [33,34] method is used to obtain the sensitive features that can differ bearing abnormal wear status. The data used in this paper mainly come from the laboratory, which has the characteristics of fewer data and small samples [35,36].

In the present work, a rotor-bearing test rig with forced lubrication was set up. The test bearing was artificially added with nonferrous contaminants with a higher hardness to simulate the abnormal wear of the bearing. Three working conditions, including normal, unbalanced, and abrasive wear are set, and multiple sensor data are collected. The ferrous particle size distribution is used to obtain effective features that can differentiate bearing wear status. The NCA method is used to extract the sensitive features from a feature set including the vibration and oil debris based-features. Finally, the SVM, KNN, and Decision Tree classification learning methods are used to analyze the importance of oil debris in the diagnosis of the bearing abnormal wear.

## 2. Feature Extraction and Dimensionality Reduction

### 2.1. Time Domain Features

The paper focuses on the fault diagnosis of bearing abnormal wear. It should be noted that for the new test bearing, before and after the entry of contaminants, the components of the bearing, including the inner ring, outer ring, rolling elements, and cage, will not be significantly damaged. Therefore, the characteristic frequency of the bearing will not change significantly. Based on this, the time domain features of vibration signals are mainly adopted. It mainly includes max value, min value, peak-to-peak value, mean, median, variance, standard deviation, mean Absolute Deviation, Skewness, Kurtosis, Crest factor, and Shape factor. The corresponding feature number is defined from No. 1 to No. 12, and the specific calculation method is shown in Table 1. Where *x*(*n*) is a discrete vibration signal, *n* = 1, 2,..., *N*, *N* is the number of sampling points of the vibration signal.

### 2.2. Time Domain Features

For supervised learning, namely the learning with the label, Neighborhood Component Analysis (NCA) is an effective dimensionality reduction method and classification learning method [34]. For a multi-classification problem with a training set containing *n* input samples, suppose its input samples are {*x*_1_, *x*_2_, *x*_3_, …, *x_n_*}, *x_i_* ∈ $R^{d}$, *i* = 1, 2, 3, …, *n*, and have corresponding labels {*y*_1_, *y*_2_, *y*_3_, …, *y_n_*}. The Mahalanobis distance between any two input samples is

(1) $$\begin{array}{l} d(x_{i},x_{j})=\sqrt{{\left(Ax_{i}-Ax_{j}\right)}^{T}(Ax_{i}-Ax_{j})}\\ \;\;\;\;\;\;\;\;\;\;\;\;\;\;=\sqrt{{\left(x_{i}-x_{j}\right)}^{T}A^{T}A(x_{i}-x_{j})} \end{array}$$

where *A^T^A* is the transformation matrix of Mahalanobis distance.

Define the probability that the sample point *x_i_* inherits the label of its close sample point *x_j_* as

(2) $$\left\{\begin{array}{l} p_{ij}=\frac{\exp(-\left\|Ax_{i}-Ax_{j}\right\|)}{\sum_{k\neq i}\exp(-\left\|Ax_{i}-Ax_{k}\right\|)},\;\;\;\;j\neq i\\ p_{ij}=0,\;\;\;\;\;\;\;\;\;\;\;\;\;\;\;\;\;\;\;\;\;\;\;\;\;\;\;\;\;\;\;\;\;\;\;j=i \end{array}\right.$$

Since any sample point can be used as a neighbor sample point, this means that the sample point *x_i_* can inherit the labels of all sample points, and the probability that the sample point *x_i_* can be correctly classified is

(3) $$p_{i}=\sum_{j\in C_{i}}p_{ij}$$

where *C_i_* represents the data set having the same class label as *x_i_*.

The search principle of NCA is to find an optimal transformation matrix *A*, which can maximize the number of sample points that are correctly classified. The corresponding objective function is defined as

(4) $$f(A)=\sum_{i}p_{i}=\sum_{i}\sum_{j\in C_{i}}p_{ij}$$

Differentiate Equation (4) to transformation matrix *A*, as

(5) $$\frac{\partial f(A)}{\partial A}=-2A\sum_{i}\sum_{j\in C_{i}}p_{ij}(x_{ij}x_{ij}{}^{T}-\sum_{k}p_{ij}x_{ik}x_{ik}^{T})=0$$

where *x_ij_* = *x_i_* − *x_j_*. According to Equation (5), the optimal transformation matrix *A* can be solved, and then the feature weight of each feature can be obtained.

### 2.3. Normalization

Normalization is a prerequisite for improving the accuracy of diagnosis. Normalization means that before the extraction of sensitive features, each feature of samples often has a different dimension. Through normalization, the feature value of each dimension is mapped to the same interval, so that each feature value has the same dimension and is in the same order of magnitude. There are mainly two commonly used normalization methods, namely Min-Max and Normalization z-score. This paper uses the former, and its specific formula is as

(6) $$v_{t}^{*}=\frac{v_{t}-\min(v_{t})}{\max(v_{t})-\min(v_{t})},\;\;t=1,2,\ldots ,T$$

where, *v_t_* is the value of the *t*-th feature, and *T* is the total number of features.

## 3. Experimental Setup

A rolling element bearing is a key component of rotating machinery. Affected by skidding, alternating loads, environmental corrosion, and electric corrosion, the bearing is prone to early failure, such as pitting and spalling. Moreover, the spalled ferrous particles will in turn accelerate the abnormal wear of the bearing. The occurrence of abnormal wear usually leads to changes in debris amount in the lubricating oil and bearing temperature. The bearing wear test in this paper is mainly for the abrasive wear caused by the entry of contaminants. With the fusion of ferrous particle data, vibration data, and temperature data, the bearing wear mechanism is studied, and then the abnormal wear is diagnosed.

**Figure 1:**
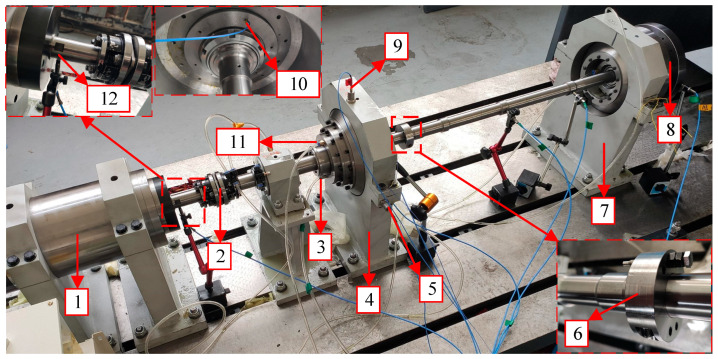
Test rig of the rolling bearing-rotor system and sensors lay out (1-motorized spindle, 2-coupling, 3-forced lubrication inlet, 4-housing containing test bearing, 5-accelerometer in the horizontal direction, 6-disc with unbalance, 7-housing containing support bearing, 8-disc, 9-accelerometer in the vertical direction, 10-thermocouple, 11-forced lubrication outlet, 12-tachometer).

**Figure 2:**
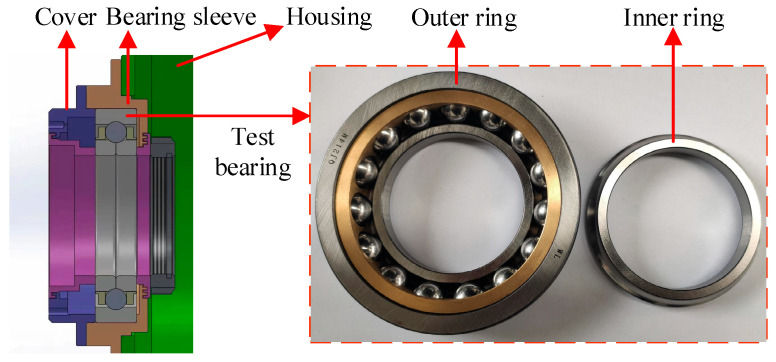
Test bearing and its installation.

**Figure 3:**
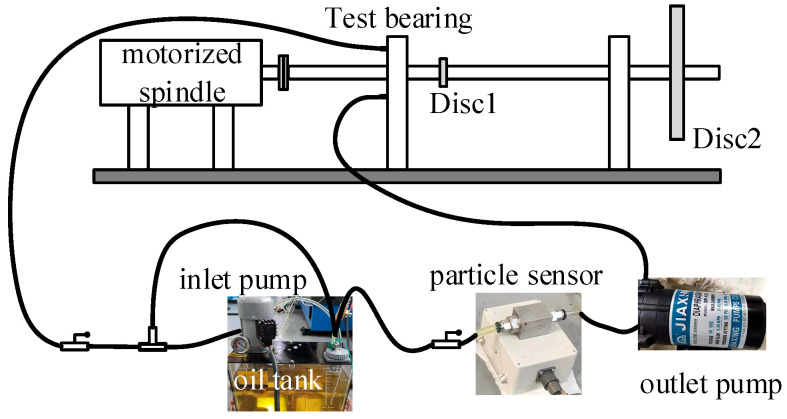
Forced lubrication system.

The rolling bearing-rotor test rig with forced lubrication is used to simulate a low pressure rotor system in an Aero-engineer, as shown in Figure 1. The electric spindle is cooled by forced water circulation. The bearing near the motorized spindle is the test bearing. The test bearing is a ball bearing with four-point contact. The bearing and its installation position are shown in Figure 2, and its specific parameters are shown in Table 2. The oil circuit of the forced lubrication system is shown in Figure 3. The lubricant type used in bearing lubrication is commercial 32# steam turbine oil and it has no antiwear agents. The flow rate is about 0.2 L/min.

The particle sensor used in this paper is an electromagnetic type ferrous particle sensor. It can detect ferrous particles as well as nonferrous particles in lubricating oil. For ferrous particles, according to the size of the particles, they are mainly divided into 10 size ranges, and nonferrous particles are mainly divided into four size ranges, as shown in Table 3. The particle is assumed to be a sphere with a diameter equal to the average particle size, which can be used to obtain the mass of the ferrous particles and nonferrous particles at different size ranges. For the health monitoring of rolling bearings, it is necessary to monitor nonferrous particles. This is because the bearing chamber may contain grinding wheel residues or carbon deposits generated in the service process, resulting in skidding damage of the bearing.

**Figure 4:**
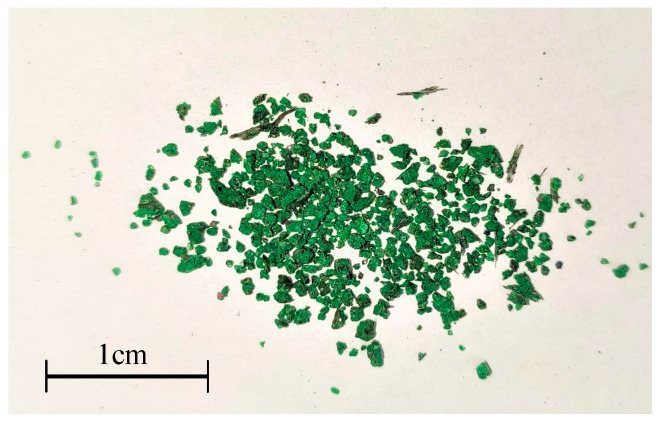
Nonferrous particle contaminants.

This paper mainly focuses on the abnormal wear caused by pitting and spalling of bearing, which is simulated by the entry of particle contaminants, which is defined as abrasive wear. Since rotor unbalances change the contact characteristics of the bearing, the unbalance is regarded as a contrast condition. The two conditions will be compared with the normal condition. The specific contents for the three conditions are shown in Table 4. The position of unbalance is shown in Figure 1, and the unbalance mass is 400 g·mm. For abrasive wear caused by the entry of contaminants, the contaminants are mainly obtained from a grinding wheel, as shown in Figure 4. The materials used for the grinding wheel are diamond and cubic boron nitride, which are nonferrous and have high hardness. The nonferrous particle contaminant is selected to be able to distinguish whether the oil debris are introduced from the outside or the bearing itself. It can be ensured that all ferrous particles detected by the electromagnetic oil debris sensor come from bearing pitting or spalling.

**Figure 5:**
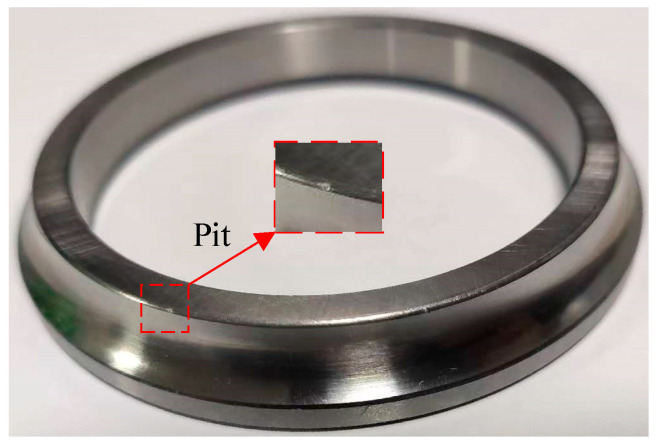
The pit on inner ring after bearing abrasive wear test.

Using the LMS data acquisition system, the horizontal and vertical acceleration of the test bearing housing and rotational speed were synchronously collected, and the sampling frequency was 5120 Hz. Simultaneously, the oil debris monitoring data and temperature data of the bearing were also synchronously collected with the sampling frequency of 0.2 Hz and 0.05 Hz, respectively. The temperature of the outer ring of the test bearing is measured by a thermocouple. The specific installation position of the thermocouple is shown in Figure 1. After the abrasive wear experiment was completed, the test bearing is disassembled. The pits can be seen on the surface of the inner ring, as shown in Figure 5, which can be used to justify the subsequent results.

## 4. Raw Data Analysis

The experimental data mainly include the horizontal and vertical vibration acceleration data of the bearing housing, the temperature data of the outer ring of the test bearing, and the oil debris data.

### 4.1. Vibration Data

**Figure 6:**
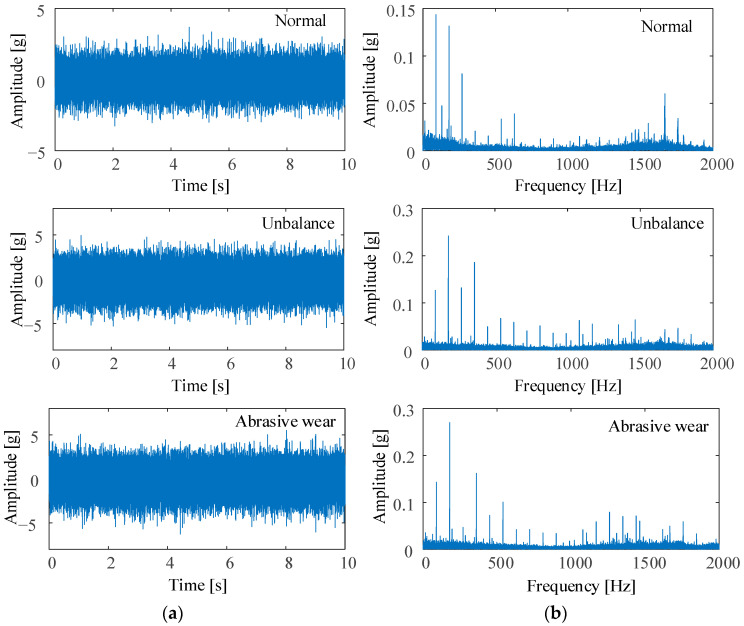
Vibration data sample at 30 min (**a**) waveform, and (**b**) envelope spectrum for the three conditions.

Take the horizontal vibration acceleration of 10 s collected when the bearing runs for 30 min under three conditions for comparison, as shown in Figure 6a. The maximum amplitude of the vibration acceleration waveform of the bearing under normal conditions is about 3 g. The maximum amplitude under unbalanced and abrasive wear conditions is about 5 g. The unbalance increases the vibration amplitude of the bearing by about 2 g. The entry of nonferrous particle contaminants does not cause significant changes in acceleration amplitude.

Envelope spectrums of vibration acceleration under three conditions are shown in Figure 6b. Under normal conditions, the operation of the new bearing exists run-in period wear. Therefore, the high-frequency component (around 1700 Hz) of the envelope spectrum at the 30-min under normal conditions is higher than that of the other two conditions. The following oil debris monitoring data analysis can also illustrate this point.

### 4.2. Temperature Data

**Figure 7:**
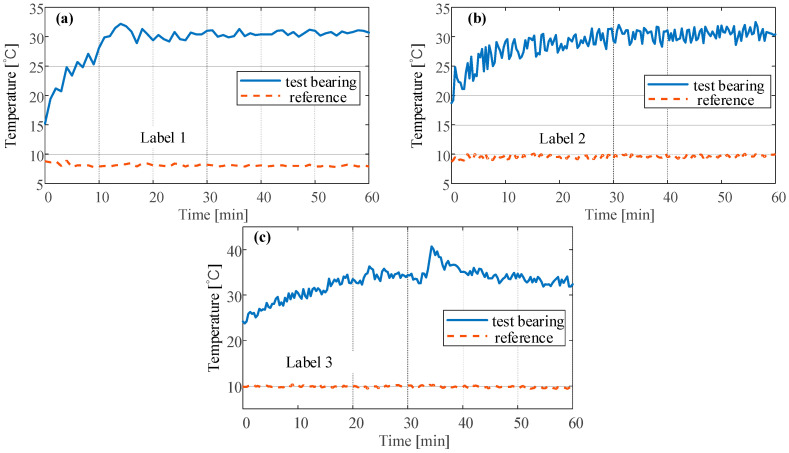
The temperature of the outer ring at (**a**) normal (**b**) unbalance (**c**) abrasive wear.

The temperatures of the outer ring under the three conditions are shown in Figure 7. The bearing temperature change at normal conditions is shown in Figure 7a, the blue solid line is the temperature of the outer ring, and the yellow dashed line is the reference temperature, namely, room temperature. As the rotational speed rises from 0 rpm to 5400 rpm, the bearing temperature rises from 15 °C to around 30 °C. After that, there is a slight increasing trend. The bearing temperature at unbalance is shown in Figure 7b, and it still rises to around 30 °C and stabilizes. The effect of unbalance on the temperature of the bearing is not obvious. However, the time required to reach a stable temperature is greater than that at normal conditions.

As shown in Figure 7c, after introducing nonferrous particle contaminants, the bearing temperature changes significantly. The temperature rises from around 25 °C to around 35 °C. Around 34 min, the bearing temperature experienced a larger fluctuation, the highest temperature reached 40 °C rapidly and then stabilized at about 35 °C. The bearing abnormal wear caused by the nonferrous particle contaminants increases the bearing temperature by about 5 °C within 2 min.

### 4.3. Oil Debris Monitoring Data

**Figure 8:**
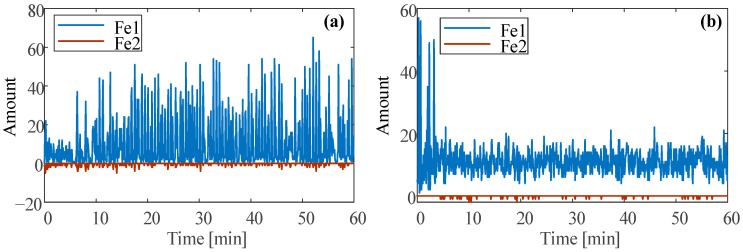
Ferrous particle amount at (**a**) normal and (**b**) unbalance.

**Figure 9:**
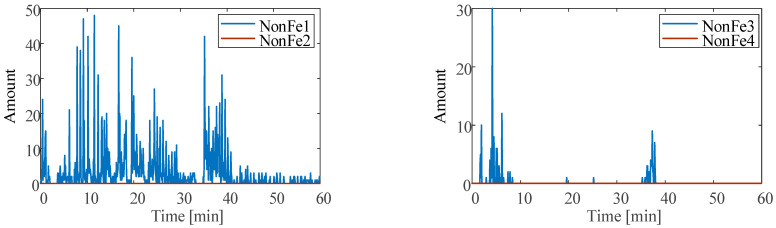
Nonferrous particles amount at abrasive wear.

**Figure 10:**
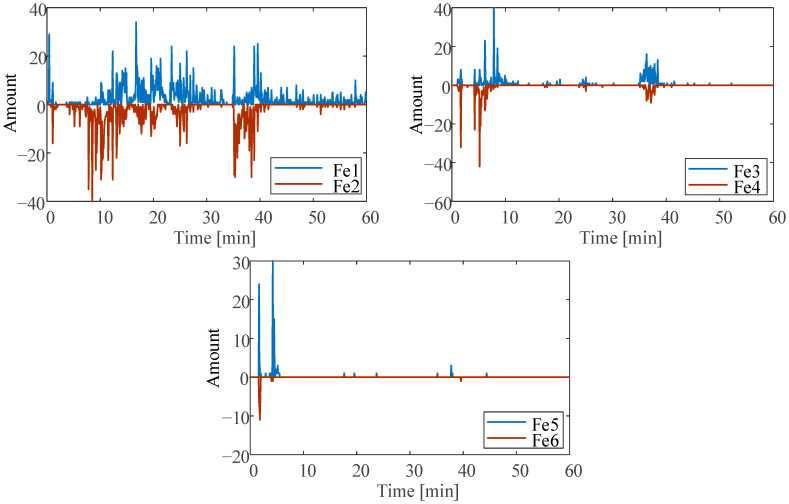
Ferrous oil debris particle amount at abrasive wear.

The particle data under the three conditions are shown in Figure 8, Figure 9 and Figure 10. There are almost no large-size ferrous particles under normal conditions and unbalanced conditions. Therefore, only the amount of ferrous particles in the Fe1 and Fe2 size ranges are considered, as shown in Figure 8.

Under normal conditions, the ferrous particles mainly come from the Fe1 size range, accompanied by a small amount of particles from the Fe2 size range. A large number of ferrous particles are less than 50 μm in size. As running time increases, the amount of particles in the Fe1 size range tends to increase slightly. The reason is that the test bearing is new, and the initial running belongs to the run-in period. This is consistent with the changing trend of the temperature at normal.

Under unbalance conditions, the ferrous particles in the Fe1 size range are still dominant, the transient amount is about 10. Compared with the normal condition, the amount is decreased. This is because the unbalance force makes the ball and the raceways evenly contact and decreases the contact instability, which makes the wear more uniform, so the amount of ferrous particles is decreased.

Under the abrasive wear condition, the amount of the nonferrous particles in the first four size ranges (NonFe1-NonFe4) are considered, as shown in Figure 9. To reproduce the pitting or spall fault of bearings, the nonferrous particle contaminants are added into the bearing during operation. The contact surfaces of the raceways and balls will rapidly be pitted or spalled through abrasive wear. Due to flow rate or adhesion, it is hard to confirm whether the contaminants enter the bearing. By monitoring the size and amount of nonferrous particle contaminants, one can accurately know the time when the contaminants contact the raceways and balls. It can be seen that nonferrous particle contaminants are mainly in the two size ranges of NonFe1 and NonFe3. Through the amount of particles in the NonFe3 size range, around 2 min, 5 min, and 37 min, there are large-size particle contaminants.

The amount of the first six ferrous particle size ranges (Fe1–Fe6) at abrasive wear is shown in Figure 10. When the nonferrous particle contaminants are introduced, the size ranges of the ferrous particles are increased. The appearance of the max amount of Fe3 (65 μm) particles is slightly behind the occurrence of the maximum amount of NonFe3. The occurrence time of the maximum amount of Fe5 and Fe6 particles is basically the same as the occurrence time of the maximum amount of NonFe3 particles. This is due to when producing pitting or spalling after nonferrous particle contaminants enter the bearing, the spalled ferrous particles will not leave the bearing immediately with the forced lubrication, but will stay in the bearing for some time. During this period, the large-size ferrous particles are crushed by the rotation of the bearing, and the particle size gradually decreases.

In addition, the amount of ferrous particles in the Fe1 and Fe2 size range is highly correlated with the number of nonferrous particles in the NonFe1 size range. The amount of ferrous particles from the Fe3 to Fe6 size range is highly correlated with the amount of nonferrous particles in the NonFe3 size range. It can be concluded that the large nonferrous particle contaminants in the NonFe3 size range will cause spalling, while small contaminants in the NonFe1 size range will only cause pitting.

At the same time, it can be found that the temperature is earlier than the oil debris signal. This is because the thermocouple directly measures the bearing temperature, but the oil debris has to pass through the forced lubrication circuit, so the oil debris signal is delayed. In addition, the above results indicate that it is necessary to detect ferrous and nonferrous particles, to determine whether the wear is caused by the spall of the bearing or the introduction of foreign contaminants.

**Figure 11:**
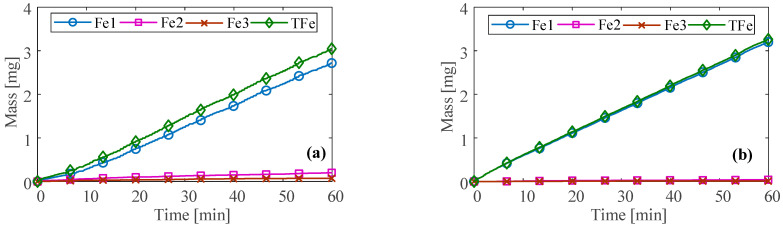
Ferrous particle accumulated mass at (**a**) normal and (**b**) unbalance.

**Figure 12:**
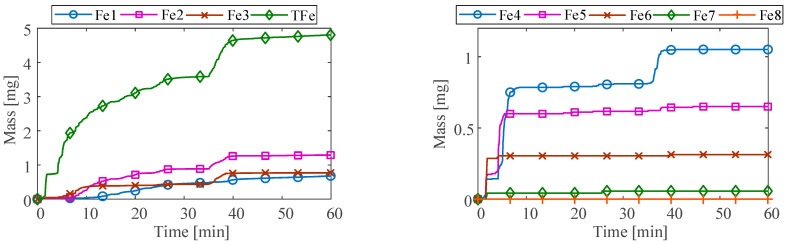
Ferrous particle accumulated mass at abrasive wear.

The different wear statuses of the bearing can affect the accumulated mass of ferrous particles. The accumulated mass of ferrous particles in each size range under the three conditions measured by the ODM and the total accumulated mass (TFe) in all size ranges over time are shown in Figure 11 and Figure 12. It can be seen that the total accumulated mass under normal and unbalanced conditions slowly increases over time, reaching about 3 mg at 60 min. The changing trend is basically the same, and mainly includes the ferrous particles in the first three sizes ranges. However, under normal conditions, the accumulated mass in the Fe2 size range is higher than that at the unbalance condition.

The accumulated mass of ferrous particles in each size range at the abrasive wear condition varies significantly compared to the previous two conditions. There are ferrous particles in the first seven size ranges. After large-size nonferrous contaminants come into contact with the bearing, the total accumulated mass fluctuates significantly. Moreover, the total accumulated mass is highly correlated with the accumulated mass in the Fe2, Fe3, and Fe4 size ranges, indicating that the ferrous debris caused by the abrasive wear mainly existed in the three size ranges.

**Figure 13:**
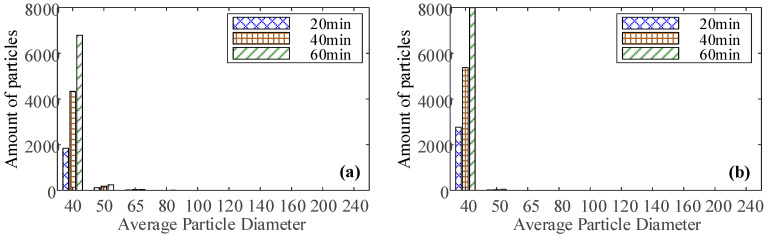

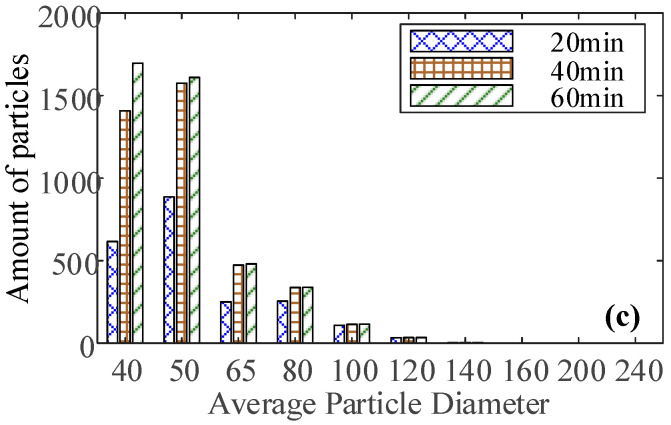
The ferrous particle size distributions at (**a**) normal, (**b**) unbalance, and (**c**) abrasive wear.

The size distributions of the accumulated mass of ferrous particles at 20 min, 40 min, and 60 min under three conditions are extracted, as shown in Figure 13. The size distributions of the accumulated mass of ferrous particles at normal and unbalanced are similar, while the size distribution at abrasive wear is significantly different, and the size range is larger.

### 4.4. Oil Debris Based-Features

Oil debris monitoring is commonly used to determine the degree of bearing wear, but this paper attempts to use oil debris monitoring to diagnose the bearing’s abnormal wear fault. For diagnosis based on oil debris monitoring, the accumulated mass of ferrous particles is often combined with a threshold to perform diagnosis. However, this method can only monitor the later stage of wear or severe wear, and can not be used for abnormal wear in the early stage. Due to the size distribution at abrasive wear being significantly different from the other two conditions, this paper proposes to extract effective features from the size distribution to judge the happen of abnormal wear.

Skewness is used to evaluate the symmetry of the distribution, and the size distribution of ferrous particles shows positive skewness under the three conditions. However, the tail on the right side of size distribution under normal and unbalanced is longer than that at abrasive wear. Skewness is mainly used to assess the weight of the tail relative to the rest of the distribution, the heavier the tail, the greater the skewness value. Therefore, the Relative Kurtosis (RK) and Relative Skewness (RS) are used to quantitatively evaluate the size distribution and are marked as No. 13 and No. 14, respectively. The specific calculation methods are defined as,

(7) $$\mathrm{Relative}\;\mathrm{Kurtosis}=\mathrm{RK}=\mathrm{Kurtosis}/{\left(\mathrm{Variance}\right)}^{2}$$

(8) $$\mathrm{Relative}\;\mathrm{Skewness}=\mathrm{RS}=\mathrm{Skewness}/{\left(\mathrm{Variance}\right)}^{3/2}$$

where, the Kurtosis, Skewness, and Variance can be obtained as

(9) $$\mathrm{Variance}=\sum_{j=1}^{N}{\left(d_{j}-E(d)\right)}^{2}P[d_{j}]$$

(10) $$\mathrm{Kurtosis}=\sum_{j=1}^{N}{\left(d_{j}-E(d)\right)}^{4}P[d_{j}]$$

(11) $$\mathrm{Skewness}=\sum_{j=1}^{N}{\left(d_{j}-E(d)\right)}^{3}P[d_{j}]$$

where *d_j_* is equal to the average bin size, *j* is equal to the number of bins and *P*[*d_j_*] is equal to the particles per average bin size divided by the total number of particles. *E*(*d*) represents the Mean particle size and can be calculated as

(12) $$\mathrm{Mean}\;\mathrm{particle}\;\mathrm{size}=E(d)=\sum_{j=1}^{N}d_{j}P[d_{j}]$$

The Relative Skewness and Relative Kurtosis of the size distribution of ferrous particles at 20 min, 40 min, and 60 min under the three conditions in Figure 13 are calculated as shown in Table 5. It can be seen that the Relative Kurtosis at unbalance is greatest and the Relative Kurtosis at abrasive wear is minimum. The Relative skewness has similar characteristics. The two feature indicators have obvious differentiation for the three conditions when at different stages and can be used to differentiate the three statuses. In addition, with the increase in operating time, both feature indicators have an increasing trend, indicating that the features can also be used to characterize the degree of wear.

## 5. Fault Diagnosis of Bearing Abnormal Wear

After obtaining all the vibration time domain features and oil debris features, the next step is to diagnose and classify the bearing wear fault. It mainly includes two parts, the first is the extraction of sensitive features, and the second is fault classification.

### 5.1. Sensitive Feature Extraction

First, use the formulas in Table 1 to obtain 12 time-domain features of the vibration signal. With the fusion of the oil debris based-features RK and RS, the features set containing 14 features is obtained. Because not all of the 12 time-domain features are sensitive to bearing wear fault. Therefore, to extract the sensitive features in the feature parameter set, after the feature parameter set is normalized, the NCA method is used for dimensionality reduction, and the weight of each feature is obtained.

**Figure 14:**
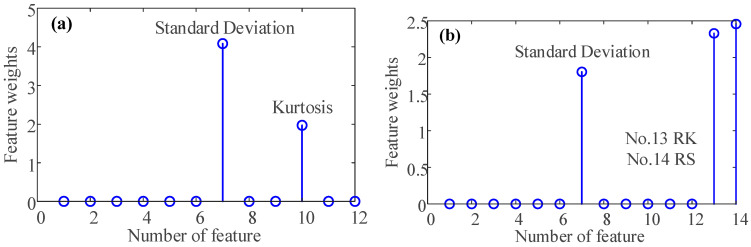
Feature weights of the sensitive feature from (**a**) feature parameter set 1 (**b**) feature parameter set 2.

**Figure 15:**
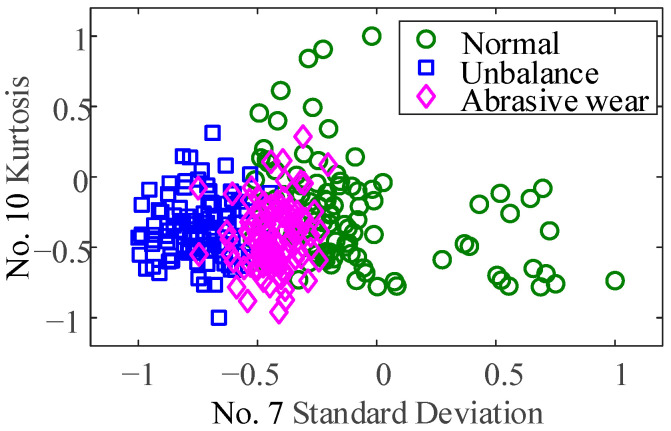
Scatter of sensitive features of standard Deviation and Kurtosis.

This paper first extracts the sensitive features in feature set 1, which only considers the 12 time domain features. It can be seen from Figure 14a that there are two sensitive time-domain features extracted by the NCA method. Sorted by feature weight, they are standard Deviation and Kurtosis, respectively. The corresponding sensitive feature distribution is shown in Figure 15. The time domain sensitive features at the different conditions are separated from each other slightly. However, the degree of differentiation is not obvious enough, and the dispersion of features under a single condition is also large, which will inevitably affect the accuracy of classification.

**Figure 16:**
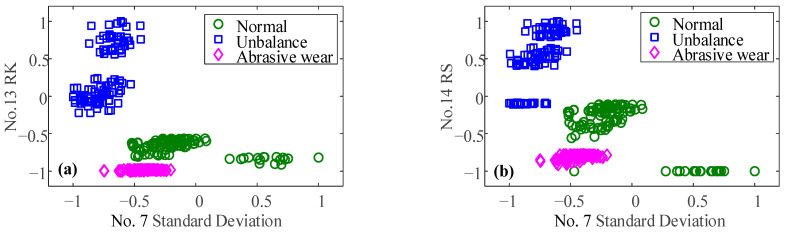
Scatter of (**a**) standard Deviation and RK (**b**) standard Deviation and RS.

To verify the importance of the oil debris based-features for the diagnosis of bearing wear faults, based on feature set 1, the oil debris based-features RK and RS are also considered. There are 14 features in total, and the feature parameter set is defined as feature parameter set 2. The NCA method is used for sensitive feature extraction and weight analysis, and the results are shown in Figure 14b. When considering the oil debris based-features, there are three sensitive features. Sorted by feature weight, they are RS, RK, and standard deviation, respectively. The distribution of the corresponding sensitive features is shown in Figure 15 and Figure 16. The particle features at different conditions are separated from each other. Therefore, particle features can effectively improve the accuracy of bearing wear fault classification.

### 5.2. The Influence of Oil Debris Feature on Classification

Based on the small data sample and tagged features of laboratory data, this paper compares the SVM, KNN, and Decision Tree classification learning methods. The influence of considering and not considering oil debris based-features on the classification accuracy are compared. Finally, the influence of the NCA dimensionality reduction methods on the result is also compared. Therefore, there are six combination methods of feature dimensionality reduction and classification learning methods used in this paper, including KNN-NCA, KNN-all features, SVM-NCA, SVM-all features, Decision Tree-NCA, and Decision Tree-all features.

**Figure 17:**
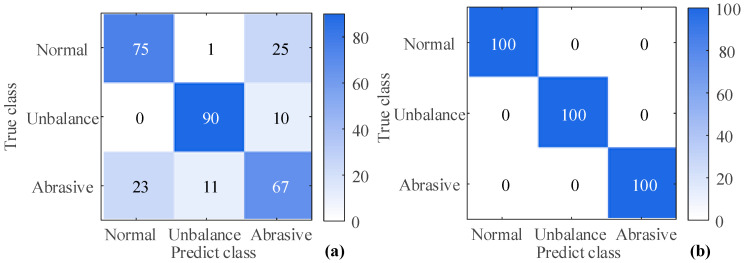
Confusion matrix using Tree-NCA method (**a**) without particles (**b**) with particles.

After the model training at different conditions is completed, a five-fold cross-validation method is adopted to obtain the classification accuracy of the trained model. The classification accuracy [37] is defined as

(13) $$Accuracy=\frac{TP+TN}{TP+TN+FP+FN}$$

where, *TP*, *TN*, *FP*, and *FN* represent the number of true positives, true negatives, false positives, and false negatives, respectively. The results of the confusion matrix and the classification accuracy are shown in Figure 17 and Table 6, respectively.

As shown in Table 6, the highest classification accuracy is 100%, which corresponds to the Decision tree methods when considering oil debris based-features. The lowest classification accuracy is 72.5%, which corresponds to the KNN-all features method without considering oil debris based-features. The accuracy of considering the oil debris based-features is significantly higher than that of not considering it. The lowest classification accuracy after considering the oil debris based-features is 94.4%, which corresponds to the KNN-all features method. When not considering oil debris based-features, the highest classification accuracy is 84%, which corresponds to the SVM methods. Therefore, considering the oil debris based-features can significantly improve the classification accuracy. The classification accuracy of the SVM methods is significantly higher than that of the KNN methods without considering the particle features. However, after considering the oil debris based-features and using NCA for dimensionality reduction, the difference in classification accuracy between the SVM and KNN methods is not obvious.

## 6. Conclusions

Based on a bearing-rotor test rig with forced lubrication, this paper carries out a mechanism study and fault diagnosis of bearing abnormal wear. Three different conditions, including normal, unbalanced, and abrasive wear caused by the entry of contaminants are considered. The contaminants will accelerate the occurrence of pitting or spalling to simulate the bearing’s early or abnormal failure. The vibration, oil debris, and temperature data of the bearing at a constant speed are collected synchronously. The results show that the abnormal wear will increase the amount of large-size ferrous particles. The large-size particle amount and temperature data are highly correlated. The oil debris based-features RK (Relative Kurtosis) and RS (Relative Skewness) are established for bearing fault diagnosis. The NCA (Neighborhood Component Analysis) method is used to extract the sensitive features. The oil debris based-features are more sensitive to the bearing wear state than the vibration based-features. Finally, three machine learning algorithms combined with NCA and all features are used to analyze the importance of the oil debris based-features on the diagnosis.

The results show that the classification accuracy with the fusion of oil debris based-features is significantly higher than that only relying on vibration time domain features. Under the feature dimension reduction using NCA, compared with that only relying on vibration based-features, the classification accuracies of the three classifiers SVM, KNN, and Decision Tree considering the oil debris-based features are increased by 15.7%, 22.2%, and 22.5%, respectively. Therefore, in order to improve the accuracy of fault diagnosis, multiple types of sensor data should be considered. Further research will focus on more in-depth extraction of real-time features of particles, such as compound detection in chromatography or spectrometry. The longer oil debris monitoring will also be carried out and the impact of historical data interception length on diagnostic accuracy will be analyzed.

## Figures and Tables

**Table 1:** Main time domain features.

Feature	Equation	No.
maximum	max(*x*(*n*))	1
minimum	min(*x*(*n*))	2
peak-to-peak value	$x_{p-p}=\max(x(n))-\min(x(n))$	3
mean	$x_{m}=\sum_{n=1}^{N}x(n)/N$	4
median	median(*x*(*n*))	5
variance	$x_{\mathrm{var}=}\sum_{n=1}^{N}{\left(x(n)-x_{m}\right)}^{2}/N$	6
standard deviation	$x_{\mathrm{std}}=\sqrt{\sum_{n=1}^{N}{\left(x(n)-x_{m}\right)}^{2}/(N-1)}$	7
mean Absolute Deviation	$x_{\mathrm{mad}}=\sum_{n=1}^{N}\left|x(n)-x_{m}\right|/N$	8
Skewness	$x_{\mathrm{ske}}=\sum_{n=1}^{N}{\left(x(n)-x_{m}\right)}^{3}/(N-1)/x_{\mathrm{std}}^{3}$	9
Kurtosis	$x_{\mathrm{kur}}=\sum_{n=1}^{N}{\left(x(n)-x_{m}\right)}^{4}/(N-1)/x_{\mathrm{std}}^{4}$	10
Crest factor	$CF=\max|x(n)|/x_{\mathrm{rms}}$	11
Shape factor	$SF=x_{\mathrm{rms}}•N/\sum_{n=1}^{N}\left|x(n)\right|$	12

**Table 2:** Parameters of the test bearing.

Parameters	Value
Bearing model	QJ214M
Inner diameter	70 mm
Outer diameter	125 mm
Width	24 mm
Ball diameter	16 mm
Ball number	15
Contact angle	35°

**Table 3:** Ferrous and nonferrous particle size ranges (average diameter, μm).

Particle Type	Bin.1	Bin.2	Bin.3	Bin.4	Bin.5	Bin.6	Bin.7	Bin.8	Bin.9	Bin.10
Ferrous	40	50	65	80	100	120	140	160	200	>240
Nonferrous	160	320	640	>1280	-	-	-	-	-	-

**Table 4:** The contents of the experiment.

Condition	Speed	Unbalance	Contaminants	Time
Normal	5400 rpm	no	no	60 min
Unbalance	5400 rpm	400 g·mm	no	60 min
Abrasive wear	5400 rpm	400 g·mm	yes	60 min

**Table 5:** Relative Skewness and Relative Kurtosis of Particle Distributions at the three conditions.

Feature	Normal			Unbalance			Abrasive Wear		
	20 min	40 min	60 min	20 min	40 min	60 min	20 min	40 min	60 min
RK	46.99	77.25	90.99	187.75	251.34	407.03	5.34	7.05	7.40
RS	5.80	7.44	8.14	12.61	14.59	17.77	1.57	1.87	1.95

**Table 6:** Classification accuracy (mean) with different methods.

Classification Learning Method	*Accuracy*	
	Without Particles	With Particles
SVM-NCA	84%	99.7%
SVM-all features	84%	99.7%
KNN-NCA	77.5%	99.7%
KNN-all features	72.5%	94.4%
Decision Tree-NCA	77.5%	100%
Decision Tree-all features	80.4%	100%

## Data Availability

The data presented in this study are available on a reasonable request from the corresponding author.
